# Regulation of Poly(ADP-Ribose) Polymerase 1 Activity by Y-Box-Binding Protein 1

**DOI:** 10.3390/biom10091325

**Published:** 2020-09-16

**Authors:** Konstantin N. Naumenko, Mariya V. Sukhanova, Loic Hamon, Tatyana A. Kurgina, Elizaveta E. Alemasova, Mikhail M. Kutuzov, David Pastré, Olga I. Lavrik

**Affiliations:** 1 Institute of Chemical Biology and Fundamental Medicine, SB RAS, 630090 Novosibirsk, Russia; k-naumenko@mail.ru (K.N.N.); sukhanovamv@mail.ru (M.V.S.); t.a.kurgina@gmail.com (T.A.K.); lisenok.istreb@gmail.com (E.E.A.); kutuzov.mm@mail.ru (M.M.K.); 2 Laboratoire Structure-Activité des Biomolécules Normales et Pathologiques, University of Evry, INSERM U1204, Université Paris-Saclay, 91025 Evry, France; loic.hamon@univ-evry.fr (L.H.); david.pastre@univ-evry.fr (D.P.); 3 Department of Molecular Biology, Novosibirsk State University, 630090 Novosibirsk, Russia

**Keywords:** Y-box-binding protein 1, poly(ADP-ribose) polymerase 1, protein poly(ADP-ribosyl)ation

## Abstract

Y-box-binding protein 1 (YB-1) is a multifunctional positively charged protein that interacts with DNA or RNA and poly(ADP-ribose) (PAR). YB-1 is poly(ADP-ribosyl)ated and stimulates poly(ADP-ribose) polymerase 1 (PARP1) activity. Here, we studied the mechanism of YB-1-dependent PAR synthesis by PARP1 in vitro using biochemical and atomic force microscopy assays. PAR synthesis activity of PARP1 is known to be facilitated by co-factors such as Mg^2+^. However, in contrast to an Mg^2+^-dependent reaction, the activation of PARP1 by YB-1 is accompanied by overall up-regulation of protein PARylation and shortening of the PAR polymer. Therefore, YB-1 and cation co-factors stimulated PAR synthesis in divergent ways. PARP1 autoPARylation in the presence of YB-1 as well as trans-PARylation of YB-1 are greatly affected by the type of damaged DNA, suggesting that PARP1 activation depends on the formation of a PARP1–YB-1–DNA ternary complex. An unstructured C-terminal part of YB-1 involved in an interaction with PAR behaves similarly to full-length YB-1, indicating that both DNA and PAR binding are involved in the stimulation of PARP1 activity by YB-1. Thus, YB-1 is likely linked to the regulation of PARylation events in cells via an interaction with PAR and damaged DNA.

## 1. Introduction

Poly(ADP-ribose) polymerases (PARPs) belong to the family of ADP-ribosyl transferase enzymes, which catalyse the synthesis of a poly(ADP-ribose) (PAR) polymer using NAD^+^ as a substrate [1,2,3]. The resulting PAR polymer is attached to an acceptor protein(s), including PARPs themselves, leading to protein poly(ADP-ribosyl)ation (PARylation) [1,2,3]. Owing to nuclear PARPs’ ability to bind directly and to be activated by damaged DNA, PARP1 and PARP2 catalyse protein PARylation during the DNA damage response [2,4,5]. These PARPs are implicated in the regulation of numerous DNA repair pathways, and PARP activation on DNA damage sites appears to have a dual function, for example, local PAR synthesis for recruitment of repair enzymes and trans-PARylation of repair factors [3,6,7,8,9]. Although PARP1 has been found to be a major acceptor of PAR during its own activation [10,11], to date, approximately 2389 proteins have been identified as PARylation targets after different types of genotoxic stress [12]. Even though numerous PARP targets have been found, many of them can both be modified by PAR and/or directly interact with the PAR synthesised by PARPs [1,8,13,14,15]. Initially, preferential targets of PARPs were shown to be PARPs themselves and DNA-binding proteins such as histones and DNA repair factors [3,8]. Later, by a mass spectrometry-based method to screen PAR-associated proteins, researchers found that numerous RNA-binding proteins can be PARylated and/or interact with PAR under genotoxic stress [14,15,16]. In a genome-wide screening intended to detect protein targets of PARPs, multifunctional Y-box-binding protein 1 (YB-1) has been identified as a substrate candidate for PARP-specific reactions [16]. YB-1 contains a cold shock domain (CSD) and was found to be a DNA-binding protein participating in the regulation of transcription in animals [17,18]. To date, YB-1 has been shown to take part in various cellular processes and to interact specifically with DNA or RNA and protein partners, such as PARP1 [19] and several DNA repair proteins including DNA polymerase β [20], NEIL-2 glycosylase [20], apurinic/apyrimidinic endonuclease 1 (*APE1*) [21], and human endonuclease III (hNTH1) [22]. Accordingly, YB-1 can be involved in both DNA and RNA metabolism depending on whether it is located in the nucleus or cytoplasm [23]. It is known that, during genotoxic stress, YB-1 is transferred from the cytoplasm to nucleus [24,25,26,27], and its interactions with damaged DNA and DNA repair proteins have been determined [19,28,29,30]. Our previous studies have revealed that YB-1 can be PARylated and functionally interacts with PARP1 [19,31,32]. Taking into account the key role of PARP1 in the regulation of DNA repair and in the maintenance of genomic stability [1,3,8,32], special attention is now given to proteins participating in the modulation of PARP1 auto- and trans-PARylation activity in vivo [33,34]. In this regard, a number of recently identified RNA-binding proteins with intrinsically disordered regions implicated in the DNA damage response because of an interaction with PAR or its PARylation also deserve research attention [35,36]. At this point, it would be interesting to investigate the regulation of PARP1 enzymatic activity by YB-1, which contains disordered domains in its structure, interacts with DNA and PAR, and is strongly PARylated in vitro [31,32]. In addition, YB-1 has nuclear localisation in the aggressive types of cancer resistant to chemotherapy and influence the sensitivity of cancer cells to anticancer drugs and the efficiency of chemotherapy [26,27,37]. Therefore, functional interplay between PARP1 and YB-1 may affect the application of PARP1 inhibitors in cancer treatment [38].

Previously, we have found that YB-1 acts as an effector of PARP1 autoPARylation, and that the unstructured positively charged C-terminal domain of YB-1 participates in PARP1 activation [32]. Additionally, we have observed that the functional interaction of YB-1 and PARP1 is affected by the presence of PAR polymers and depends on the formation of a ternary PARP1–YB-1–DNA complex [32]. Therefore, in the present study, we further examined the interplay between recombinant YB-1 and PARP1 in vitro by biochemical and atomic force microscopy (AFM) assays to characterise the protein PARylation and PAR synthesis and to elucidate the molecular mechanism underlying the YB-1-mediated regulation of PARP1 activity. The influence of wild type and mutant YB-1 on the PARylation activity of PARP1 was evaluated by considering a broad spectrum of substrates, including DNA duplexes, mononucleosomes, and plasmid DNA. It turned out that PARP1 auto- and trans-PARylation activities in the presence of YB-1 are affected by the type of DNA, suggesting that PARP1 activation in the presence of YB-1 is dependent on the formation of the PARP1–YB-1–DNA ternary complex. The disordered C-terminal domain of YB-1 as well as full-length YB-1 were capable of stimulating PARP1 activity and of being PARylated, suggesting that the C-terminal domain is essential for the YB-1-dependent up-regulation of PARP1 activity. PARP1 activity was also tested in the presence of YB-1 and divalent or multivalent cations or small positively charged proteins (histones), which are required for efficient catalytic activity of PARP1 and have a potential for binding to both DNA and PAR molecules. The PAR synthesis activity of PARP1 turned out to be highly dependent on the nature of the co-factor. Namely, in contrast to an Mg^2+^-dependent reaction, YB-1-dependent PARP1 activation was accompanied by an increase in the overall level of protein modification and shortened chains of PAR during autoPARylation. Addition of exogenous YB-1 to a nuclear extract enhanced the overall PAR synthesis, probably owing to both PARP auto-PARylation and YB-1 trans-PARylation, in agreement with the effect observed in the systems with recombinant proteins. To sum up, stimulation of PARP1 activity by YB-1 depends not only on its interaction with DNA but also on its ability to interact with PAR likely via the disordered C -terminal domain during the reaction of PARP1 auto-PARylation in ternary complex PARP1–YB-1–DNA, resulting in YB-1 trans-PARylation and an increase in overall efficiency of protein PARylation.

## 2. Materials and Methods

### 2.1. Proteins and Reagents

Yeast nicotinamide mononucleotide adenylyltransferase (NMNAT), APE1, and core histones (H2A, H2B, H3, and H4) purified from chicken erythrocytes were kindly provided by Dr. S.I. Shram (IMG, the Russian Academy of Sciences (RAS), Moscow, Russia) and Dr. S.N. Khodyreva (the Institute of Chemical Biology and Fundamental Medicine (ICBFM), the Siberian Branch of the Russian Academy of Sciences (SB RAS), Novosibirsk, Russia), respectively. A histone H1 and bovine serum albumin (BSA) were purchased from Sigma (Sigma-Aldrich, Saint–Louse, MO, USA). Core histones (H2A, H2B, H3, and H4) were purified from chicken erythrocytes as described in [39]. The fragment of YB-1 containing an alanine/proline-rich N-terminal domain and the cold shock domain (AP-CSD) was а kind gift from Drs. L.P. Ovchinnikov and D.A. Kretov (Institute of Protein Research RAS, Pushchino, Russia). Recombinant Escherichia coli uracil-DNA glycosylase (UDG) was acquired from Biosan (Novosibirsk, Russia). Nuclear extracts from HeLa cells were prepared as described elsewhere [40]. Protein concentration in the extracts was determined as described in [41], with BSA as a standard. Plasmid pET-3-1-YB-1 containing human *YB-1* cDNA was a generous gift from L.P. Ovchinnikov and D.A. Kretov. PCR products containing the full YB-1-coding sequence, a sequence encoding a truncated form of YB-1 (containing amino acid residues 1–219) or a sequence encoding the C-terminal domain of YB-1 (CTD fragment) were cloned into the pLate-51 plasmid vector (ThermoFisher Scientific, Waltham, MA, USA). The sequences of the resultant proteins were confirmed at the SB RAS Genomics Core Facility (ICBFM SB RAS, Novosibirsk, Russia). Each recombinant protein (YB-1, YB-1(1-219), or the CTD fragment) was overexpressed in *E. coli* BL21(DE3) and purified. Namely, YB-1 was purified by Ni-NTA affinity chromatography (GE Healthcare, Chicago, IL, USA), Mono-S chromatography (GE Healthcare), and Superdex 200 chromatography (GE Healthcare) as described earlier [42]. YB-1(1-219) was purified by Ni-NTA and Mono-S chromatography, and the CTD fragment by Ni-NTA chromatography. The plasmid DNA containing human *PARP1* cDNA was a kind gift from Dr. M. Satoh (Université Laval, Québec, Canada). Mutation E988K within the PARP1 coding sequence was generated by site-directed mutagenesis with Q5-polymerase (New England Biolabs, Ipswich, MA, USA). The sequence of the resulting protein was confirmed at the SB RAS Genomics Core Facility. Recombinant PARP1 and PARP1(E988K) were purified as described previously [43]. [α^32^P]ATP (3000 Ci/mmol) was produced in the Laboratory of Biotechnology, ICBFM SB RAS. NAD^+^ and β-nicotinamide mononucleotide were bought from Sigma (Sigma-Aldrich). The oligodeoxynucleotides used in this work were synthesised by Biosset (Novosibirsk, Russia) and the Laboratory of Biomedical Chemistry (ICBFM SB RAS, Novosibirsk, Russia). Schematic structures of these oligodeoxynucleotide duplexes are shown in Table 1.

### 2.2. Preparation of DNA Duplexes and Plasmid DNA

DNA duplexes were obtained by hybridisation of complementary oligonucleotides in a 1.0:1.5 ratio. Oligonucleotide mixtures were incubated for 5 min at 95 °C and then slowly cooled to room temperature. To prepare the pBR plasmid containing one-nucleotide gaps, the plasmid DNA (0.25 mg/mL) was incubated at 70 °C for 45 min in a buffer consisting of 20 mM sodium citrate (pH 5.0) and 200 mM NaCl [44]. The reactions were stopped by the addition of 1 M Tris-HCl (pH 8.0); then, the apurinic/apyrimidinic-site–containing pBR plasmid (29 nM) was incubated with 20 nM APE1 in a buffer consisting of 50 mM NaCl, 50 mM Tris-HCl (pH 8.0), and 5 mM MgCl_2_ for 1 h at 37 °C. The reaction mixture was subjected to heat inactivation for 10 min at 65 °C, rapidly chilled on ice, and used for subsequent reactions. The circular forms of plasmid DNA containing one-nucleotide gaps with 3′-OH and 5′-deoxyribose phosphate (dRP) were analysed by 0.8% agarose gel electrophoresis (Figure S1).

### 2.3. Preparation of Mononucleosome Substrates

To prepare 147 bp DNA, PCR was carried out under the following conditions: a denaturation step (95 °C for 3 min); 5 amplification cycles (94 °C for 15 s, 65 °C for 15 s (with a decrement of 1 °C per cycle from 65 °C to 60 °C), and 72 °C for 10 s); 25 amplification cycles (94 °C for 15 s, 60 °C for 15 s and 72 °C for 10 s). The plasmid DNA template containing nucleosome positioning sequence 603 at 1 ng/µL was supplemented with 0.5 µM primers (forward: 5′-ACCCCAGGGAC**U**TGAAGTAATAAGG-3′, reverse: FAM-5′-CCCAGTTCGCGCGCCCACC-3′) and standard PCR buffer components (Biolabmix, Moscow, Russia). It should be noted that one of the primers contained uracil at the +6 position from the 5′ end and 6-carboxyfluorescein (FAM) at the 5′ end (Table 2). To remove the excess of primers, the samples were treated with exonuclease ExoI. The PCR product (147 bp DNA) was ethanol precipitated and dissolved in double-distilled H_2_O.

Mononucleosomes were reconstituted by the salt-dialysis method, as described earlier [39]. Briefly, an appropriate histone/DNA ratio was found experimentally by the quick-time reconstitution approach. The 147 bp DNA at a final concentration of 0.1 µM was mixed with histones of different concentrations in low-salt buffer (10 mM NaCl, 0.2 mM EDTA, 5 mM β-mercaptoethanol, 0.1% of NP-40, and 10 mM Tris-HCl pH 7.5), incubated for 15 min at 37 °C, and analysed by 4% polyacrylamide gel electrophoresis (PAGE) under non-denaturing conditions (Supplementary Figure S2A). The lowest DNA/histone ratio corresponding to a mixture containing no naked 147 pb DNA was chosen for preparative mononucleosome reconstitution. DNA and histones in an appropriate ratio were mixed in a high-salt buffer containing 2 M NaCl and dialysed for 6 h at 4 °C against the buffer with a gradient of NaCl from 2 M to 250 mM with gentle stirring. Then, the probes were dialysed against the buffer with 10 mM NaCl overnight at 4 °C with gentle stirring and analysed by 4% PAGE under non-denaturing conditions (Supplementary Figure S2B). Concentrations of the resulting mononucleosomes were determined by measuring absorbance at 260 nm.

For preparation of damaged 147 bp DNA or a mononucleosome, the reaction mixture (total volume of 10 μL) consisted of reaction buffer (50 mM Tris-HCl pH 8.0, 40 mM NaCl, and 1 mM DTT) and either 1 µM reconstituted nucleosome or 1 µM 147 bp DNA. The reaction components were mixed on ice. The reaction was initiated by the addition of UDG and EndoIII to a final concentration of 50 and 20 nM, respectively. The mixture was incubated at 37 °C for 15 min. The resultant gapped 147 bp DNA or mononucleosomes were employed to test PARP1 activity (Table 2).

### 2.4. A Radioactive Assay of Protein PARylation by PARP1

[^32^P]NAD labelled on the adenylate phosphate was synthesised as described earlier [31]. The protein PARylation assay was performed in the reaction mixtures consisting of 50 mM Tris-HCl pH 8.0, 40 mM NaCl and 1 mM DTT; 5 mM MgCl_2_, 10 mM EDTA or 2 mM spermine; either 30 nM PARP1 or 100 nM PARP1 (E988K); 1600 nM YB-1 (or one of its mutants), 0.054 mg/mL mixture of chicken erythrocyte core histones (H2A, H2B, H3, and H4), or 0.054 mg/mL recombinant histone H1; and 100 nM DNA duplex, 3.5 nM plasmid DNA, 0.5 OD_260_/mL activated DNA, 100 nM 147 bp DNA, or 100 nM mononucleosome. The mixtures were incubated at 37 °C for 15–60 min.

To test PARP activity in the HeLa nuclear extract, the reactions were allowed to proceed in mixtures (total volume 10 μL) consisting of 50 mM Tris-HCl pH 8.0, 40 mM NaCl, 1 mM DTT, 0.25 OD_260_/mL activated calf thymus DNA, 1.0 mg/mL cell extract proteins, 5 mM MgCl_2_, 1 µM PARG inhibitor, and 1600 nM YB-1 where indicated. The mixtures were incubated for 10 min at 37 °C.

The reaction components were mixed on ice. All the reactions were initiated by adding NAD^+^ (0.4 μCi [^32^P]NAD^+^) to a final concentration of 4 or 400 μM. The reactions were stopped either by adding SDS sample buffer and heating for 1.5 min at 97 °C or by placing the samples drop-wise on Whatman 1 paper filters preimpregnated with 10% trichloroacetic acid (TCA). Then, the reaction mixtures were analysed by denaturing PAGE as described elsewhere [45] with modifications. Briefly, a step gradient separating 4% and 10% gels (70:1 acrylamide/bisacrylamide ratio, pH 8.8) was used. The PARylated proteins were visualised by phosphor imaging by means of Typhoon FLA 7000 (GE Healthcare, Chicago, IL, USA). In the case of TCA precipitation, the aliquots spotted onto the Whatman filter paper impregnated with 10% TCA were washed four times with 5% TCA and then 96% EtOH. Then, the filter paper pieces were dried, and TCA-insoluble radioactivity (PARylated proteins) was measured by radioautography using Typhoon FLA 7000 (GE Healthcare).

### 2.5. AFM Experiments and Image Analysis

For experiments with the autoPARylation of PARP1, 35 nM PARP1 was incubated with 3.5 nM gapped pBR in a buffer (12.5 mM HEPES, pH 8.0, 12.5 mM KCl, and 1 mM DTT) in the presence or absence of 560 nM YB-1 (or its fragment called AP-CSD) and/or 10 mM MgCl_2_. The reactions were initiated by the addition of NAD^+^ to a final concentration of 400 μM followed by incubation for 1 h at 37 °C. Next, the samples were diluted 10-fold with AFM deposition buffer (12.5 mM HEPES, pH 8.0, 12.5 mM KCl, and 1 mM DTT) and immediately deposited on a mica surface. For AFM imaging, the samples were processed as described previously [46]. The mica surface was then rinsed with a 0.02% uranyl acetate solution, rapidly rinsed with pure water (Millipore), and air-dried before AFM imaging in ambient air [47] by means of Nanoscope V Multimode 8 (Bruker, Santa-Barbara, CA, USA) in peakforce tapping (PFT) mode with Scanasyst-Air probes (Bruker). In this experiment, continuous force–distance curves were recorded at 2048 × 2048 pixels at a line rate of 1.5 Hz, and the tip was oscillated in the vertical direction with an amplitude of 100–300 nm at a low frequency (1–2 kHz). The sizes of PARylated proteins were determined in the ImageJ software and calculated using the following equation: S = π·R_min_·R_max_, where R_min_ and R_max_ are semi-major and semi-minor axes, respectively, of the minimal ellipse in which a PARylated protein could be enclosed.

### 2.6. Fluorescence Anisotropy Measurements

Reaction mixtures consisting of a buffer (50 mM Tris-HCl pH 8.0, 40 mM NaCl, and 1 mM DTT), 100 nM PARP1, 1600 nM YB-1, 10 mM EDTA, and one of four substrates (100 nM undamaged 147 bp DNA, damaged 147 bp DNA with a one-nucleotide gap, an undamaged mononucleosome, or a damaged mononucleosome with a one-nucleotide gap) were prepared on ice in Corning black 384-well polystyrene assay plates and incubated for 5 min at room temperature. The fluorescent probes were excited at 482 nm (482–16 filter plus dichroic filter LP504), and the fluorescence intensity was detected at 530 nm (530–40 filter). Each measurement consisted of 50 flashes per well, and the obtained fluorescence values were automatically averaged. The measurement was done in kinetic scan mode. The measurements of each well were carried out 72 times at intervals of 50 s. To analyse the binding of either PARP1 or PARP1 and YB-1 to either 147 bp DNA or a mononucleosome, fluorescent anisotropy values were determined before NAD^+^ addition. To analyse dissociation of PARP1 and YB-1 from the complexes with either 147 bp DNA or a mononucleosome during PARylation, the reaction mixtures were supplemented with NAD^+^ to a final concentration of 4 μM after the first cycle. The data were processed in the MARS Data Analysis Software (BMG LABTECH GmbH, Germany). All the measurements were conducted in duplicate for each specific condition and were performed at least three times.

### 2.7. Statistical Analysis

All the experiments were conducted at least three times. The quantitative data were analysed in Microsoft Excel, version 14.0.7258.5000 (Microsoft Corporation, Redmond, WA, USA, 2010) and presented in histograms as the mean ± SD.

## 3. Results

### 3.1. Stimulation of PARP1 Activity by YB-1 is Affected by the Type of DNA Damage

**Figure 1:**
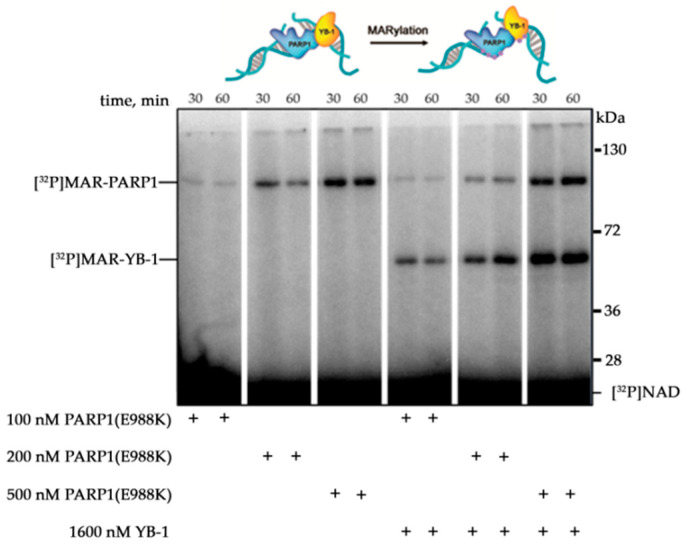
Y-box-binding protein 1 (YB-1) stimulates mono(ADP-ribosyl)ation (MARylation) activity of the poly(ADP-ribose) polymerase 1 (PARP1)(E988K) mutant. Evaluation of protein MARylation in the presence of [^32^P]NAD and DNA duplexes containing a nick by SDS-polyacrylamide gel electrophoresis (PAGE) with subsequent phosphor imaging. The reaction mixtures contained 100–500 nM PARP1(E988K), 100 nM DNA substrate 4 μM NAD^+^ and [^32^P]NAD^+^ (0.4 μCi), and 1600 nM YB-1, as indicated.

We have previously reported that YB-1 can stimulate poly(ADP-ribose) (PAR) synthesis and PARP1 autoPARylation in the absence of divalent cations, which act as co-factors for PAR synthesis [19,32]. YB-1 is a multifunctional protein and can interact with DNA, RNA, and PAR [42,48]. Thus, two possible mechanisms have been proposed to explain the regulation of PARP1 activity by YB-1: direct interaction of YB-1 either with damaged DNA or with PAR polymer synthesised by PARP1 [32]. Nevertheless, the details of the mechanism behind YB-1-dependent stimulation of PARP1 activity have *not* been determined. Our previous study has shown that YB-1 interacts with PAR, and that this interaction can contribute to PARP1 activity in the presence of YB-1 [32]. Here, we first tested whether YB-1 stimulates the PARP1(E988K) mutant [49], which manifests mono(ADP-ribosyl)ation (MARylation) activity (Figure 1).

**Figure 2:**
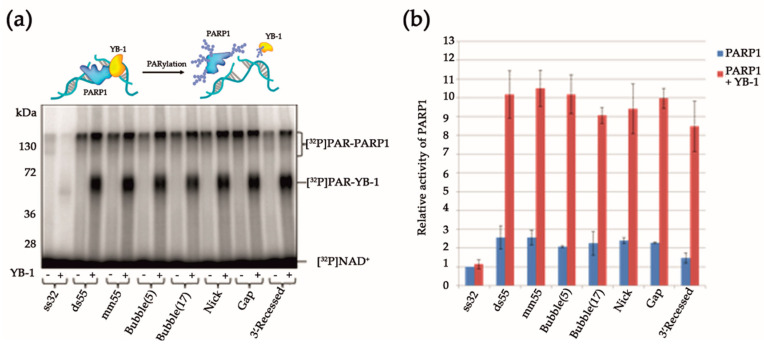
YB-1 stimulates PARP1 activity in the presence of different types of *DNA* damage. (**a**) Protein PARylation in the presence of DNA duplexes and [^32^P]NAD according to SDS-PAGE with phosphor imaging. The reaction mixtures contained 100 nM PARP1, 100 nM DNA substrate 4 μM NAD^+^ and [^32^P]NAD (0.4 μCi), and 1600 nM YB-1, as indicated. (**b**) Quantification of PARP1 activity. PARP1 at 100 nM was incubated with 100 nM DNA substrate, and 4 μM NAD^+^ and [^32^P]NAD (0.4 μCi) in the presence of 1600 nM YB-1, as indicated; ^32^P-PAR–modified proteins were trichloroacetic acid (TCA)-precipitated and counted. The relative level of PAR synthesis was normalized to the level of PAR synthesis catalyzed by PARP1 alone for 15 min with ss32 DNA substrate. The experiments were conducted at least three times; the histogram shows the means ± SD of three independent experiments.

YB-1 was found to stimulate the activity of PARP1(E988K), leading to MARylation of both proteins. Thus, the stimulation of MAR synthesis catalysed by the PARP1 mutant was documented in the case of attachment of one ADP-ribose moiety to PARP1 and YB-1 (Figure 1). This result suggests that up-regulation of PARP1 activity by YB-1 can take place in the absence of a PAR polymer. These data support the notion that the formation of the PARP1–YB-1–DNA ternary complex is important for PARP1 activity stimulation. Therefore, it is possible that the effect of YB-1 on PARP1 activity depends on the type of damaged DNA. To clarify this issue, the levels of PAR synthesis and protein PARylation were assessed in the presence of YB-1 using various DNA structures that induce PARP1 activation (single- or double-strand breaks or mismatched nucleotides) and/or serve as substrates preferred by YB-1 such as bubble-type duplexes or single-stranded DNA (ssDNA) (Table 1). For ssDNA called ss32, the presence of YB-1 led to a slight effect on PARP1 activity, suggesting that YB-1 binds to ssDNA more effectively than PARP1 does, and the formation of the YB-1–DNA–PARP1 ternary complex is suppressed in this case. In the presence of double-stranded DNA duplexes containing damage, YB-1 stimulated PAR synthesis 3.5–5-fold, which was accompanied by abundant PARylation of both proteins (Figure 2a,b).

The formation of PARP1–DNA–YB-1 ternary complexes seems to be an important stage of PARylation reactions catalysed by PARP1; therefore, the intensity of the protein modification depends on the interaction of PARP1 and YB-1 in their ternary complex with DNA. Indeed, we observed some differences in the ratio of the levels of YB-1 and PARP1 modification depending on the type of DNA structure used, but PARylation of YB-1 was slightly more efficient than that of PARP1 for these DNA substrates (Figure 2a). Furthermore, it was noted that the stimulation of PARP1 activity by YB-1 is only moderately affected by the type of lesion (a single-strand break, one-nucleotide gap, “bubbles” of 5 and 17 nucleotides or a mismatched base pair); this phenomenon may be a consequence of preferential activation of PARP1 by a blunt end of DNA duplexes (Figure 2b). Nonetheless, for all the structures (except ssDNA) used in the experiments, the PAR synthesis in the presence of YB-1 was notably more active, and YB-1 was the preferential target of PARylation (Figure 2a,b).

**Figure 3:**
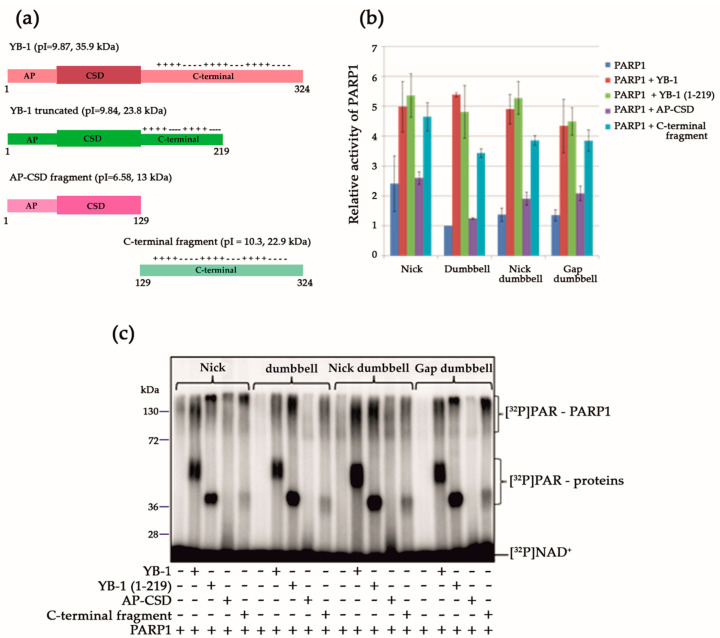
The C-terminal fragment of YB-1 is sufficient to stimulate PARP1 activity. (**a**) Schematic view of the YB-1 constructs employed in this study. (**b**) Quantification of PARP1 activity. PARP1 (100 nM) was incubated with 100 nM DNA substrate, and 4 μM NAD^+^ and [^32^P]NAD (0.4 μCi) in the presence of 1600 nM YB-1 or its mutant (where indicated). ^32^P-PAR–modified proteins were TCA-precipitated and counted. The relative level of PAR synthesis was normalized to the level of PAR synthesis catalyzed by PARP1 alone for 15 min with dumbbell DNA substrate. The experiments were performed at least three times; the histogram presents the means ± SD of three independent experiments. (**c**) Protein PARylation in the presence of DNA duplexes and [^32^P]NAD according to SDS-PAGE and phosphor imaging. The reaction mixtures contained 100 nM PARP1, 1600 nM YB-1 or its mutant (where indicated), 4 μM NAD^+^ and [^32^P]NAD (0.4 μCi), and 100 nM DNA substrate. AP-CSD, alanine/proline-rich N-terminal domain and the cold shock domain.

Further assessment of the effect of YB-1 on PARP1 activity was performed on dumbbell DNAs. The use of hairpin structures prevents PARP1 activation at the ends of DNA duplexes and clarifies the influence of other types of DNA damage such as single-strand breaks [50]. For this purpose, dumbbell undamaged (dumbbell) DNA, DNA containing a nick (nick-dumbbell), or DNA containing a one-nucleotide gap (gap-dumbbell) was used in further experiments (Table 1). Indeed, PARP1 is activated on dumbbell DNAs (Supplementary Figure S3), but less intensively as compared with blunt-ended DNA duplexes (Figure 3b).

At the same time, the impact of PARP1 stimulation by YB-1 was also pronounced in the case of dumbbell DNAs, and PAR synthesis was more active (3- to 5-fold) in the presence of YB-1 (Figure 3b). This finding implies that the formation of the PARP1–YB-1–DNA ternary complex and trans-PARylation of YB-1 occurs in the case of dumbbell DNA carrying nick or one nucleotide gap.

The ability of YB-1 to interact with DNA, RNA, PAR, and proteins seems to be related to its structural features [48,51]. This protein contains a disordered Ala/Pro-rich N-terminal domain (A/P domain), a CSD, and a disordered C-terminal domain containing clusters of positively and negatively charged amino acid residues (Figure 3a). Both the CSD and C-terminal domain of YB-1 have been reported to interact with DNA and RNA; in addition, the C-terminal domain plays a critical part in YB-1 oligomerization and in its interaction with other proteins [23,48,52]. Electrostatic interaction is a driving force behind the complex formation between the CSD or C-terminal domain with negatively charged nucleic acids such as ssDNA and ssRNA [52]. Taking into account that PAR is a negatively charged polymer, it appears that these domains can interact with the PAR synthesized by PARP1. Earlier, we have proposed that the C-terminal domain of YB-1 mediates its ability to stimulate PARP1 activity because PARP1 activation was affected only slightly in the presence of a YB-1 deletion mutant lacking this domain [32]. To further address the involvement of YB-1 domains in the regulation of PARP1 activity, an assay of protein PARylation and PAR synthesis was performed in the presence of AP-CSD (a truncated form of YB-1 lacking the C-terminal domain), the C-terminal fragment (a truncated form of YB-1 lacking AP-CSDs), or the fragment called YB-1(1-219) (a truncated form of YB-1 lacking residues 220–324 of the C-terminal domain) (Figure 3a). Dumbbell DNAs and the nicked DNA duplex were utilized in these experiments (Table 1). PARP1 activity was only slightly affected by the presence of AP-CSD (Figure 3b), whereas the C-terminal fragment stimulated PARP1 auto-PARylation and was PARylated (Figure 3b,c). Considering that the C-terminal domain of YB-1 plays a critical role in PAR binding [42], we can conclude that YB-1′s ability to modulate PARP1 activity is due to its direct interaction with PAR. Thus, the stimulation of PARP1 activity by YB-1 is associated with both the formation of YB-1–PARP1–DNA ternary complexes (Figure 1) and YB-1 binding to PAR.

### 3.2. YB-1 Modulates PARP1 Activation in the Presence of a Mononucleosome 

**Figure 4:**
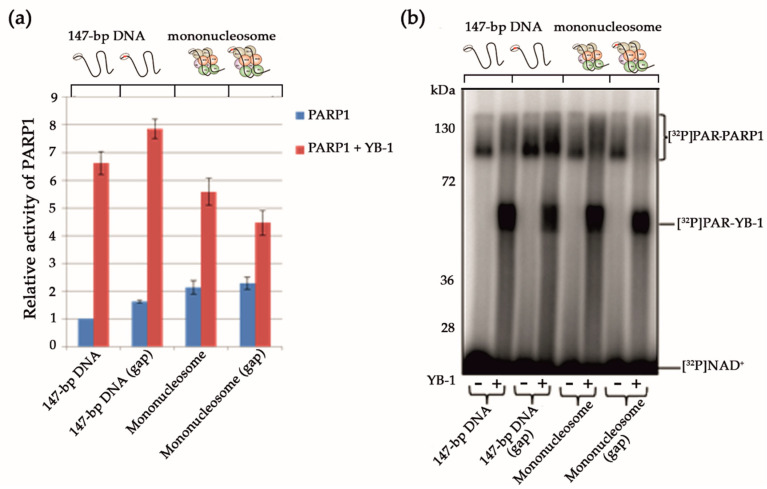
YB-1 stimulates PARP1 upon activation by a mononucleosome. (**a**) Quantification of PARP1 activity. PARP1 at 100 nM was incubated with 100 nM substrate and 4 μM NAD^+^ and [^32^P]NAD (0.4 μCi) in the presence of 1600 nM YB-1 (where indicated). ^32^P-PAR–modified proteins were TCA-precipitated and counted. The relative level of PAR synthesis was normalized to the level of PAR synthesis catalyzed by PARP1 alone for 60 min with 147 bp DNA. The experiments were conducted at least three times; the histogram shows the means ± SD of three independent experiments. (**b**) Assessment of the protein PARylation in the presence of 147 bp DNA or mononucleosome substrates and 4 μM NAD^+^ and [^32^P]NAD (0.4 μCi) [^32^P]NAD^+^ by SDS-PAGE and phosphor imaging. The reaction mixtures contained 100 nM PARP1, 1600 nM YB-1 (where indicated), and 100 nM substrate.

In eukaryotic cells, DNA is organised in a chromatin context, where the nucleosome is the main structurally repeating unit [53]. The nucleosome is a DNA–protein complex in which DNA is wrapped around a core histone octamer consisting of two copies of H2A, H2B, H3, and H4 [54]. Given that the function of PARP1 is tightly associated with chromatin in vivo and can be regulated through PARP1–histone core interactions [3,8,55,56], we tested whether YB-1 modulates PARP1 activity when mononucleosomes served as substrates. For these experiments, a mononucleosome containing either undamaged 147 bp DNA or damaged 147 bp DNA with a one-nucleotide gap (or free undamaged 147 bp or damaged 147 bp DNAs with a one-nucleotide gap as a control) was used (Table 2). We observed that YB-1 presence increases overall PAR synthesis for any of the four above-mentioned substrates (Figure 4a).

In the case of mononucleosomes, YB-1 trans-PARylation was predominantly detectable, whereas stimulation of PARP1 automodification by YB-1 was clearly detectable only in the context of free damaged 147 bp DNA (Figure 4b). It should be noted that the lowest level of PARylation of both proteins was observed in the case of PARP1 activation on the damaged nucleosome (Figure 4a). Accordingly, we cannot rule out the possibility that the PARP1 interaction with YB-1 in the context of a nucleosome is influenced by the histone core, particularly H3 and H4, which, along with DNA, are also binding targets of PARP1 and may regulate PARP1 activity [55,56,57]. These data suggested that the activity of PARP1 is regulated by YB-1 in the context of a nucleosome, but the histone core of nucleosomes influences the process.

**Figure 5:**
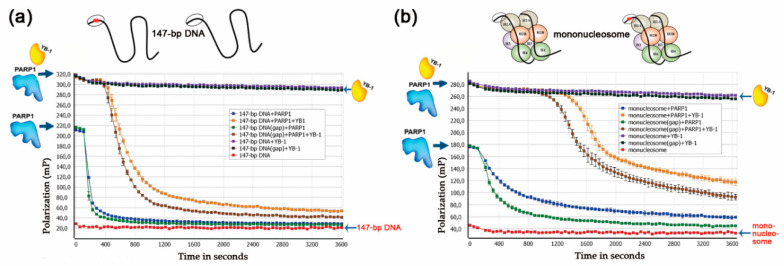
YB-1 modulates the dissociation of the autoPARylated PARP1 from 147 bp DNA substrates and mononucleosomes. (**a**) Comparative analysis of dissociation curves of the protein–DNA complex after protein PARylation in the presence of a 147 bp DNA substrate. The reaction mixtures contained 100 nM PARP1, 4 μM NAD^+^, 1600 nM YB-1 (where indicated), and 100 nM undamaged or damaged 147 bp DNA (gap). (**b**) Comparative analysis of dissociation curves of the protein–DNA complex after protein PARylation in the presence of a mononucleosome substrate. The reaction mixtures contained 100 nM PARP1, 4 μM NAD^+^, 1600 nM YB-1 (where indicated), and 100 nM undamaged or damaged mononucleosome (gap).

PARP1 activation by a mononucleosome in the presence of YB-1 was also studied by the fluorescent polarisation method adapted to real-time measurement of PARP activity; the latter method is based on the detection of dissociation of PARylated PARP1 and other proteins from DNA [58]. Numerous studies have shown that PARylation of a protein decreases its DNA-binding activity and facilitates dissociation from DNA [1,4,5,46,58,59]. To monitor the dissociation of DNA–protein complexes, we utilised fluorescein-labelled 147 bp DNAs or mononucleosomes assembled from this DNA and analysed changes in the anisotropy after the addition of proteins in the absence or presence of NAD^+^ (Figure 5).

When PARP1 with or without YB-1 was added to the substrates, we detected an increase in the anisotropy level (maximum anisotropy) owing to the binding of PARP1 alone or of both proteins to DNA (Figure 5). The addition of YB-1 to a mixture of PARP1, 147 bp DNA, and NAD^+^ changed the shape of the ‘dissociation’ curve for PARP1, suggesting that PARP1 activation in the presence of YB-1 is accompanied by slowing of the dissociation of the protein–DNA complex via PARylation (Figure 5a). This finding is in agreement with the low efficiency of PARP1 automodification in the presence of 147 bp DNA and YB-1, as observed in the gel assay of protein PARylation, because YB-1 PARylation was efficient (Figure 4b). In the case of 147 bp DNA containing a gap, intensive PARylation of both YB-1 and PARP1 (Figure 4b) was seen, causing more effective protein dissociation from DNA (Figure 5a). Similar data were obtained in the experiments on PARP1 activation in the presence of mononucleosomes and YB-1, where the addition of YB-1 to the reaction decreased autoPARylation of PARP1 on damaged mononucleosomes (Figure 4b), thus delaying protein dissociation from DNA (Figure 5b). Note that the PARP1 release from DNA during PARylation was greatly affected by the presence of YB-1, namely, this process became relatively slow and included an initial lag visible in all the experiments (Figure 5a,b). This lag varied between 200 and 400 s in the case of naked DNA and reached 600 s with mononucleosomes. Consequently, YB-1—being the preferential target of modification—affects PARP1 autoPARylation and retards PARP1 dissociation from DNA (Figure 5).

These results further support the idea that YB-1 functions as a regulator of DNA damage-dependent PARP1 autoPARylation by acting as a stimulator of PAR synthesis on a broad spectrum of DNA structures including nucleosomes.

### 3.3. Effects of Mg^2+^, Spermine, Histones, and YB-1 on PARP1 Activity In Vitro

**Figure 6:**
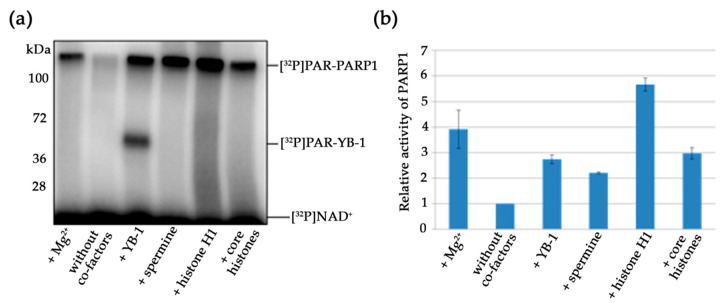
A comparison of the effects of cations, histones, and YB-1 on PARP1 activity. (**a**) Protein PARylation in the presence of different co-factors and [^32^P]NAD according to SDS-PAGE with subsequent phosphor imaging. The reaction mixtures contained 100 nM PARP1, 0.5 OD_260_/mL activated DNA, 10 mM EDTA, 5 mM Mg^2+^, 4 μM NAD^+^ and [^32^P]NAD (0.4 μCi), 1600 nM YB-1, 2 mM spermine^3+^, and 0.054 mg/mL mixture of core histones (H2A, H2B, H3, and H4) or recombinant histone H1, as indicated. (**b**) Quantification of PARP1 activity. PARP1 at 100 nM was incubated with 0.5 OD_260_/mL activated DNA, 4 μM NAD^+^ and [^32^P]NAD (0.4 μCi) in the presence of 5 mM Mg^2+^, 1600 nM YB-1, 2 mM spermine^3+^, and 0.054 mg/mL mixture [of either core histones (H2A, H2B, H3, and H4) or recombinant histone H1] or 10 mM EDTA, as indicated. ^32^P-PAR–modified proteins were TCA-precipitated and counted. The relative level of PAR synthesis was normalized to the level of PAR synthesis catalyzed by PARP1 alone for 15 min in the presence of EDTA. The experiments were conducted at least three times; the histogram presents the means ± SD of three independent experiments.

Numerous proteins related to DNA or RNA metabolism modulate PARP1 activity, and some of them can stimulate its autoPARylation [35,60,61,62]. Although Mg^2+^ is the divalent cation that is required as a co-factor for PARPs in PAR synthesis [63], other divalent and multivalent cations such as Ca^2+^ and natural polyamines (putrescine^2+^, spermine^3+^, or spermidine^4+^) can function in these reactions [61]. Along with the cations, small positively charged proteins such as histones H1, H3, and H4 can stimulate PARP1 activity [56,60,61,64,65]. As mentioned above, YB-1 stimulated PARP1 activity and its autoPARylation (Figure 2 and Figure 3). We have previously proposed that YB-1 stimulates PARP1 activity in a histone-like manner [32]. For example, similarly to histones H1 and H3 [61,64], YB-1 can stimulate PARP1 activity, and the effect is dependent on the DNA/PARP1/YB-1 ratio [32]. A molar excess of YB-1 relative to DNA and PARP1 is reported to strongly enhance PAR synthesis and YB-1 PARylation [32]. Nevertheless, the mechanism of PARylation stimulation in the presence of YB-1 is still being determined. To further examine the stimulation of PARP1 by YB-1 and to compare the influence to that seen with other co-factors of PARP1 such as Mg^2+^, spermine^3+^, recombinant histone H1, or mixture of histone core octamers (H2A, H2B, H3, and H4), we analysed PARP1 activity using the above co-factors and activated DNA (Figure 6). 

The stimulation of PARP1 by YB-1 on the activated DNA seemed to be weaker than that seen with either the histones or Mg^2+^ (Figure 6a). According to the obtained data, the co-factors that can stimulate PARP1 activity can be ranked as follows: H1 > Mg^2+^ > octamers ≥ YB-1 ≥ spermine^3+^, which is where YB-1 is PARylated (Figure 6b). Quantification of the data indicated that both YB-1 and histones enhance the PARP1 autoPARylation activity, thus pointing to similarities between histone- and YB-1-mediated PAR syntheses. Although we observed that YB-1, just as histones, stimulates the PARP1 autoPARylation, only YB-1 was strongly PARylated under these conditions (Figure 6a). Therefore, the impact of YB-1 on PAR synthesis includes two components: changes in the level of PARP1 autoPARylation and the high level of YB-1 trans-modification.

### 3.4. YB-1 Decreases the Size of the PAR Polymer Produced by PARP1 

**Figure 7:**
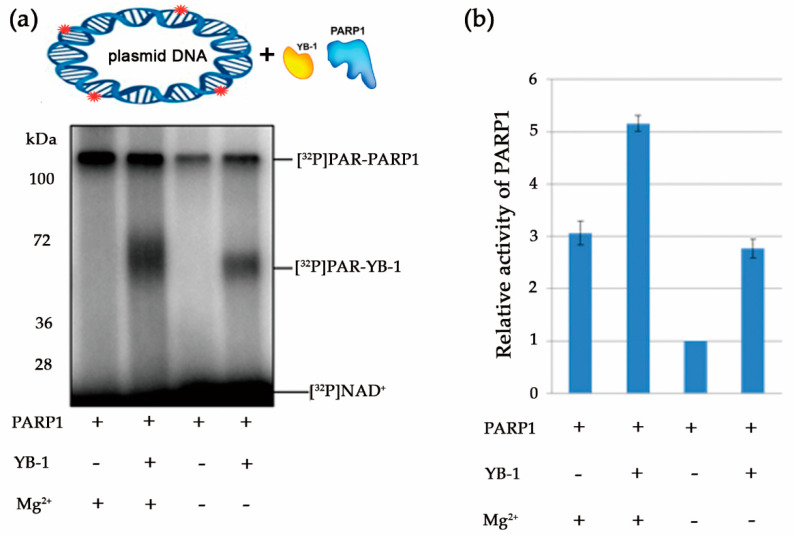
YB-1 stimulates PARP1 activity in the presence of damaged plasmid DNA. (**a**) Protein PARylation in the presence of damaged plasmid DNA and [^32^P]NAD according to SDS-PAGE and phosphor imaging. The reaction mixtures contained 30 nM PARP1, 4 μM NAD^+^ and [^32^P]NAD (0.4 μCi) 560 nM YB-1, 3 nM plasmid DNA, and 10 mM EDTA or 5 mM MgCl_2_, as indicated. (**b**) Quantification of PARP1 activity. PARP1 (30 nM) was incubated with 3 nM plasmid DNA, 4 μM NAD^+^ and [^32^P]NAD (0.4 μCi) in the presence of 560 nM YB-1, and either 10 mM EDTA or 5 mM MgCl_2_, as indicated. ^32^P-PAR–modified proteins were TCA-precipitated and counted. The relative level of PAR synthesis was normalized to the level of PAR synthesis catalyzed by PARP1 alone for 60 min without co-factors. The experiments were performed at least three times; the histogram shows the means ± SD of three independent experiments.

To further delineate the features of PARP1-catalysed reactions in the presence of YB-1, we compared the effects of YB-1 and divalent cations (Mg^2+^) on PAR synthesis, with estimation of the molecular size of the PAR polymers. The protein PARylation reactions were analysed at the single-molecule level by AFM to determine PAR polymer size and structure [46]. To avoid problems with morphological identification of a PAR polymer versus DNA duplexes on a mica surface, we employed long DNA, namely circular plasmid DNAs (containing one-nucleotide gaps), as substrates to induce PARP1 activation (Supplementary Figure S1). First, by means of the plasmid DNA, we analysed PARP1 activity in the presence or absence of Mg^2+^, YB-1, or both (Figure 7).

**Figure 8:**
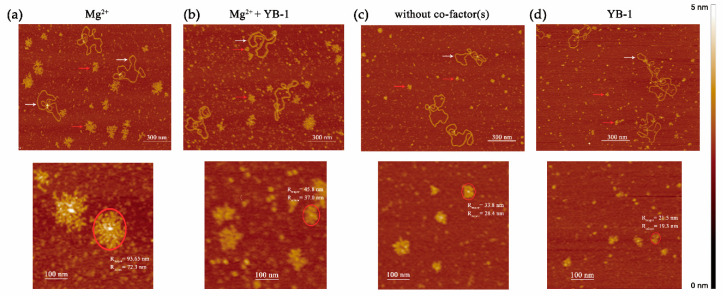
Atomic force microscopy (AFM) images of PARylated PARP1 in the presence of different co-factors. The images illustrate auto-PARylation of PARP1 in the presence of Mg^2+^ (**a**), in the presence of Mg^2+^ and YB-1 (**b**), without co-factors (**c**), or in the presence of YB-1 (**d**). Upper panel: AFM images of PARylated PARP1. White arrows indicate plasmid DNA molecules, and red arrows point to PARylated proteins. Scale bar: 300 nm; Z scale: 5 nm. Lower panel: zoomed-in images of PARylated PARP1. The two radii of the ellipse enclosing PARylated PARP1 were used to estimate the area of a modified protein molecule. Scale bar: 116 nm; Z scale: 5 nm.

Similar to DNA duplexes (Figure 2 and Figure 4), YB-1 stimulated PARP1 activity and was PARylated in the presence of damaged plasmid DNA (Figure 7). As reported elsewhere, PARP1′s co-factors, for example, Mg^2+^ and histone H1, influence both overall protein activity and average chain length of the PAR polymer [61,65]. Consequently, YB-1 could influence not only the amount of PARylated proteins (Figure 7A), but also the size of PAR polymers formed, particularly during PARP1 autoPARylation. To clarify this issue, we visualised PARylated proteins by AFM [46]. To evaluate PARylation of PARP1 in the absence of co-factors or in the presence of YB-1, Mg^2+^, or both, AFM imaging of PARP1 was undertaken after incubation with gapped pBR in the presence of NAD^+^ (Figure 8).

**Figure 9:**
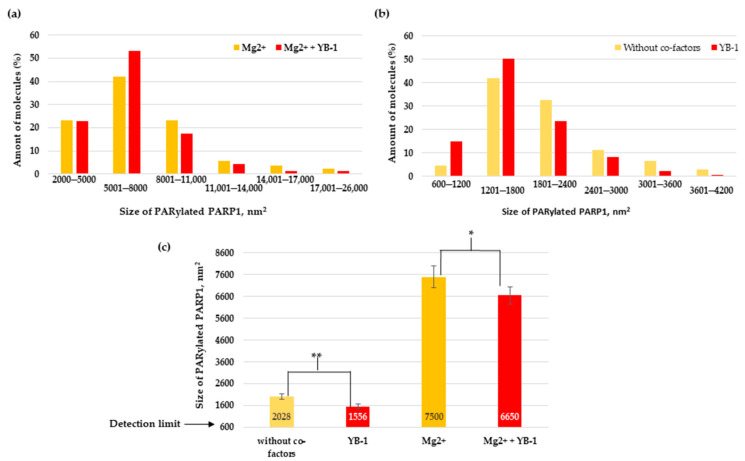
AFM-based analysis of PARylated PARP1 revealing molecular size distributions in the presence of Mg^2+^ (**a**), in the presence of Mg^2+^ and YB-1 (**a**), without Mg^2+^ (**b**), or in the presence of YB-1 without Mg^2+^ (**b**). The percentage histograms present the percentage of PARylated molecules in each size range in the presence of Mg^2+^, in the presence of Mg^2+^ plus YB-1, without co-factors, or in the presence of YB-1. Numbers of PARylated molecules analysed: 91 in the Mg^2+^ group, 81 in group ‘Mg^2+^ and YB-1′, 133 in the group without co-factors, and 145 in the YB-1 group. An ellipse with the smallest area whose centre coincided with the centre of a PARylated molecule and completely enclosed it was chosen to estimate the area of the molecules. The sizes of PARylated PARP1 smaller than 600 nm^2^ were disregarded. (**c**) The average size of PARylated PARP1 measured in images shown in (**a**,**b**). Results are mean ± SD of two to five images of three independent samples for each assay group. *p*-values were obtained by comparing the results by Student’s t-test, *, *p* < 0.05; **, *p* < 0.01.

When PARP1 was incubated with DNA in the presence of NAD^+^, the formation of PAR polymers was detectable by AFM in all reaction systems (Figure 8). The sizes of the PARylated PARP1 molecules were estimated by means of the AFM images obtained for four reaction systems: with Mg^2+^, YB-1, both, or without them (Figure 9).

The relative size distributions of PARylated molecules under the above conditions are presented in Figure 9. For Mg^2+^-dependent PAR synthesis, the PARylated molecules had a size up to 26,000 nm^2^ (Figure 9a), whereas in the absence of Mg^2+^, it was only up to 4200 nm^2^ (Figure 9b). In the case of Mg^2+^-dependent PAR synthesis, the size distribution of the PARylated molecules was slightly asymmetric and skewed towards larger sizes, and the average size was ~7500 nm^2^ (Figure 9a,c). In the YB-1-dependent reaction (Figure 9b), we also observed a positively skewed distribution; however, the molecular size of PARylated PARP1 differed from that observed with Mg^2+^ alone or in the presence of both Mg^2+^ and YB-1 (Figure 9a). As illustrated in Figure 9c, the average size was ~1556 nm^2^, which is at least 4.8-fold less than the size of the automodified PARP1 observed in the presence of Mg^2+^ (~7500 nm^2^). In the absence of both co-factors, the size distribution was also slightly asymmetric and skewed towards larger sizes of PARylated molecules; the average size was ~2028 nm^2^ (Figure 9b,c). Thus, in the YB-1-dependent reactions and in the absence of co-factors, the average size of modified PARP1 was much smaller than the size after PARylation in the presence of Mg^2+^: 2028 and 1556 versus 7500 nm^2^, respectively (Figure 9). Although Mg^2+^ alone strongly enhanced the synthesis of a long PAR chain, the addition of YB-1 to the Mg^2+^-dependent system inhibited the polymer chain growth, and we documented an apparent decrease in the average size of modified PARP1 from 7500 to 6650 nm^2^ (Figure 9c).

**Figure 10:**
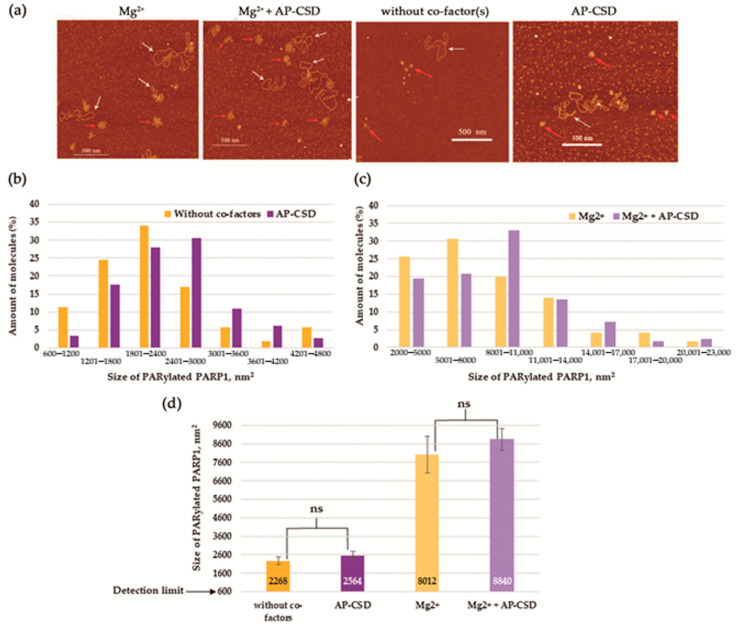
AFM-based analysis of PARylated PARP1 showing the molecular size distributions in the presence of the AP-CSD fragment. (**a**) AFM images show auto-PARylation of PARP1 in the presence of Mg^2+^, in the presence of Mg^2+^ and AP-CSD, without co-factors, and in the presence of AP-CSD. White arrows indicate plasmid DNA molecules and red arrows indicate PARylated proteins. Scale bar: 500 nm; Z scale: 5 nm. (**b**,**c**) The percentage histograms showing the percentage of PARylated molecules in each size range in the presence of Mg^2+^ (**c**), in the presence of Mg^2+^ and AP-CSD fragment (**c**), without co-factors (**b**), and in the presence of AP-CSD fragment (**b**). Number of PARylated molecules analyzed: 89 for Mg^2+^, 84 for Mg^2+^ and AP-CSD fragment, 82 for without co-factors, and 93 for AP-CSD fragments. The area of minimum ellipse enclosing the PARylated protein was used to estimate the area of PARylated proteins. The size of PARylated PARP1 smaller than 600 nm^2^ was not taken into account. (**d**) The average size of PARylated PARP1 measured in images shown in (**a**). Results are mean ± SD of two to five images of three independent samples for each assay group. *p*-values were obtained by comparing the results by Student’s *t*-test, ns, not significant.

As demonstrated above (Figure 3), the fragment called AP-CSD affected PARP1 activity only slightly (Figure 3b); moreover, the fragment was not PARylated (Figure 3c). The sizes of the PARylated PARP1 molecules were also estimated by means of the AFM images obtained for reaction systems with AP-CSD (Figure 10).

Similar to YB-1, AP-CSD affected the size distribution of PARylated PARP1, but did not induce a decrease in the size of PARylated PARP1 (Figure 10b,c). In contrast to full-length protein, the size distribution of the PARylated molecules in the presence of AP-CSD was still slightly asymmetric, but shifted more towards larger sizes of molecules (Figure 10b,c). At the same time, the values of the average size of PARylated molecules measured in the presence of AP-CSD were not significantly different from the values measured in the absence of the fragment alone or AP-CSD plus Mg^2+^ (Figure 10d).

Therefore, the presence of C-terminal YB-1 domain (Figure 3b) appears to play an important part in the regulation of PARP1 activity, in particular affecting not only the trans-PARylation of YB-1 (Figure 3), but also the size of PAR polymers produced by PARPs. This phenomenon may be a consequence of the interaction of YB-1 with the PAR polymer and may prevent its elongation by PARP1.

In summary, these data suggest that Mg^2+^-dependent PAR synthesis catalysed by PARP1 is characterised by the production of long-chain molecules. In contrast, YB-1-dependent PAR synthesis is accompanied by overall stimulation of PARP1 activity, but shortening of the produced PAR polymers. It should be noted that the effect of YB-1 was abrogated by the deletion of its disordered C-terminal domain containing acidic/basic clusters. Consequently, YB-1 modulates PARP1 activity as a positively charged protein co-factor and acceptor of PAR during protein PARylation.

### 3.5. YB-1 Modulates PAR Synthesis Activity in the HeLa Cell Extract 

**Figure 11:**
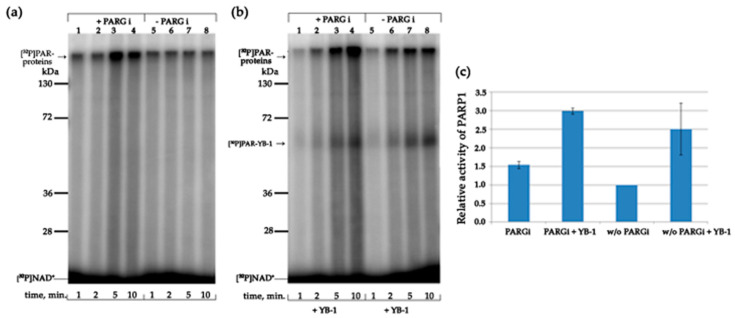
YB-1 affects the PARylation reactions catalysed by proteins of nuclear extracts from HeLa cells. (**a**,**b**) Protein PARylation in the presence of HeLa nuclear extracts and [^32^P]NAD according to SDS-PAGE and phosphor imaging. The reaction mixtures consisted of 1.0 mg/mL nuclear extract proteins, 0.25 OD_260_/mL activated DNA, 5 mM MgCl_2_, 4 μM NAD^+^ and [^32^P]NAD (0.4 μCi), 1600 nM YB-1 (**b**), and either 1 µM PARG inhibitor ((**a**,**b**), lanes 1–4) or no such inhibitor ((**a**,**b**) lanes 5–8), as indicated in the figure. The arrow points to the border between the concentrating and separating gel. (**c**) The efficiency of PAR synthesis in the nuclear extracts of HeLa cells in the presence of exogenous YB-1. Nuclear extract proteins (1.0 mg/mL) were incubated with 0.25 OD_260_/mL activated DNA, 5 mM MgCl_2_, 4 μM NAD^+^ and [^32^P]NAD (0.4 μCi), 1600 nM YB-1, and 1 µM PARG inhibitor (PARGi), as indicated in the figure. ^32^P-PAR–modified proteins were TCA-precipitated and counted. The relative level of PAR synthesis was normalized to the level of PAR synthesis catalyzed by cell extracts for 10 min in the absence of PARGi. The experiments were conducted at least three times; the histogram shows the means ± SD of three independent experiments.

To investigate the potential relevance of our in vitro findings further, we tested whether purified YB-1 modulates activation of PARPs in HeLa nuclear extracts. To examine this possibility, we analysed PARPs’ activities when Mg^2+^ alone or together with YB-1 was added to the nuclear extract (Figure 11).

Protein PARylation is known to be regulated by PARPs and poly(ADP-ribose) glycohydrolase (PARG) [66]. Degradation of PAR is catalysed by PARG, which hydrolyses the PAR polymer, thereby generating free ADP-ribose residues [67]. To avoid PAR degradation due to PARG activity of protein extracts, the protein PARylation in the nuclear extracts was studied in the presence of a PARG inhibitor: PDD 00017273 (PARGi in Figure 11). First, in the extract, we compared the Mg^2+^-dependent protein PARylation between the presence and absence of the PARG inhibitor (Figure 11a). The products of this reaction looked like smeared bands owing to variation of the length of the PAR polymer attached, and most of the PARylated proteins had a high molecular weight, over 130 kDa, and turned out to be concentrated at the border between the concentrating and separating gels (Figure 11a). As expected, the amount of the PARylated proteins in the reaction was significantly lower in the absence of the PARG inhibitor (Figure 11a, compare lanes 1–4 and 5–8). The addition of YB-1 to the extract proteins stimulated both PAR synthesis (Figure 11c) and the formation of high-molecular-weight products of PARylation (Figure 11a,b). Besides, incubation of the protein extract with YB-1 in the presence of [^32^P]NAD^+^ revealed that exogenous YB-1 is PARylated in the presence or absence of the PARG inhibitor (Figure 11b). At the same time, YB-1 addition to the extract led to the formation up-shifting smears because of the greater length of the PAR polymer attached to proteins (Figure 11b). This effect may be a consequence of both stimulation of protein PARylation by YB-1 in the extract and protection of the PAR polymer from PARG-dependent degradation, owing to the interaction of exogenous YB-1 with PAR [42].

Thus, the patterns of YB-1-driven modulation of PAR synthesis observed in the system reconstituted from purified proteins are similar to the patterns observed in the nuclear extract. These observations further support the hypothesis that YB-1 may cooperate with PARPs and participate in the regulation of PARylation events in the cell.

## 4. Discussion

By means of multifunctional RNA-binding protein YB-1, as an example, our study provides insights into the regulation of PARP1 activity by protein partners. PARP1 catalytic activity is affected by numerous factors such as cations [61], the type of damaged DNA [68,69], proteins that bind to the damaged DNA, PARP1, and/or PAR, influencing the PARylation process [34]. The PAR synthesis reaction is an unusual enzymatic process because PARPs catalyse PAR synthesis and are simultaneously acceptors of the reaction product, that is, the PAR polymer. In DNA damage-dependent PAR synthesis catalysed by PARP1 or PARP2, the negatively charged DNA serves as an activator of these PARPs [4,5,6,50,69] and a factor influencing PAR chain growth because of electrostatic repulsion between the DNA and negatively charged PAR bound to the protein [1,5]. PARP1 activity strongly depends on the presence of divalent or multivalent cations such as Mg^2+^, Ca^2+^, putrescine^2+^, or spermine^4+^; they are thought to shield the negative charge of PAR and DNA via complexation of their phosphate groups, thereby resulting in the synthesis of long branched PAR polymers during autoPARylation [61,64]. It was initially found that small positively charged proteins, such as histones, can stimulate PARP1 activity in the absence of cations [60,61,64]. In contrast to cations, the influence of proteins on PARP1 activity is more complicated because they can serve as PAR acceptors during a trans-PARylation reaction [35,61,62]. Many proteins can potentially affect PARP activity through a direct interaction with DNA or PAR polymers, which act as a scaffold molecule and facilitate the formation of a complex between PARPs and proteins [1,8,70,71]. These PARP1–protein interactions mediated by DNA or PAR mainly cause suppression of PARP1 autoPARylation, often associated with enhanced PARP1 trans-modification activity towards the interacting proteins [72,73]. Nevertheless, several chromosomal proteins, such as histones [61,64] and HMGN1 [62], can stimulate the auto-modification of PARP1. Much attention is now focused on the function of RNA-binding proteins in the regulation of PARPs’ activities because RNA-binding proteins can be PARylated and regulate PARP1 activity via an interaction with PAR [35]. One of these, YB-1, was reported to interact with damaged DNA, PARP1, and PAR and to stimulate PARP1 activity, and can be a target of PARylation [32]. YB-1 has a CSD (a putative DNA(RNA)-binding motif) and a disordered C-terminal domain with clusters of positively charged amino acid residues. This structural organisation allows multifunctional YB-1 to interact with DNA, RNA, PAR, and proteins [23,42,51,52]. This observation suggests that YB-1 may influence PARP1 activity under genotoxic stress, leading to YB-1 nuclear translocation independently from proteolytic cleavage of YB-1 by the 20S proteasome [26]. In addition, YB-1 is overexpressed in various types of cancer [27,74,75]. Consequently, it is important to understand the mechanism of action of PARP1-regulatory proteins, such as YB-1, owing to their possible influence on the effects of PARP inhibitors as anticancer drugs [32,38].

In this study, we characterised the interaction of PARP1 with YB-1 in in vitro reconstituted systems to clarify the biochemical features of PARP1 stimulation by YB-1. It has been shown previously that YB-1 can stimulate PARP1 activity in the absence of cation co-factors [32]. Here, we revealed that YB-1-dependent stimulation of PARP1 activity takes place on different types of damaged DNA, including those in the context of a nucleosome. It was demonstrated that the formation of ternary complex PARP1–YB-1–DNA is important for YB-1-mediated stimulation of PARP1 activity. The parameters of PAR synthesis that were enhanced by the presence of cations (Mg^2+^) and YB-1 were compared, and a substantial difference in the size of PAR polymers was documented (Figure 8 and Figure 9), even though both Mg^2+^ and YB-1 stimulate PARP1 activity (Figure 6). It was found that Mg^2+^-dependent PAR synthesis is characterised by the production of long-chained and branched polymers (Figure 8 and Figure 9). Although YB-1 stimulated PARP1 activity, thereby increasing the overall level of PARP1 autoPARylation, the presence of YB-1 caused shortening of the PAR during PARP1 automodification (Figure 9). Additionally, we studied the effect of separate YB-1 domains on PARP1 activity and observed that the C-terminal fragment plays a crucial role in the PARP1 stimulation by YB-1. Although YB-1 is considered a DNA/RNA-binding protein [48,52], the presence of the CSD similar to the OB fold may contribute to PAR binding because the OB fold is regarded as one of ‘PAR reader modules’ [70]. In the case of YB-1, however, we noticed that the YB-1 fragment called AP-CSD has only a moderate impact on molecular size distributions of PARylated PARP1 (Figure 10), but this fragment is not PARylated and barely affects the PARP1 activity (Figure 3). In contrast, the C-terminal fragment of YB-1 alone was found to behave similarly to full-length YB-1 or YB-1(1–219) (i.e., YB-1 with a truncated C terminus; Figure 3), suggesting that the C-terminal domain mainly takes part in YB-1-mediated PARP1 activity regulation. It has been reported that the disordered C-terminal domain of YB-1 not only contributes to its binding to DNA and protein–protein interactions, but also is necessary for YB-1 interaction with PAR [23,32,42]. The key role of intrinsically disordered domains in protein interaction with PAR and PARylation dependent regulation was demonstrated for p53 as well as for other PAR binding proteins [76].

Finally, when nuclear extracts were supplemented with YB-1, the PAR synthesis was up to twofold more active and YB-1 was PARylated despite the presence of numerous proteins (in the extract) that may participate in the modulation of PARPs’ activities and contribute to PAR metabolism. Thereby, YB-1 is likely linked to the regulation of PARylation events in cells via an interaction with PAR and damaged DNA, notably in cancer cells when YB-1 is located in the nucleus.

## 5. Conclusions

**Figure 12:**
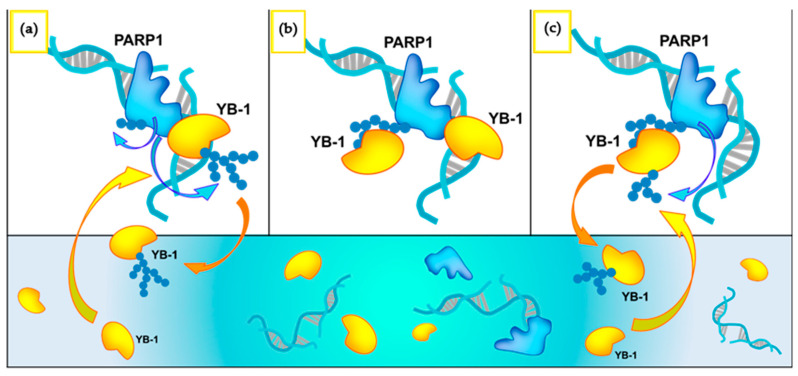
A simplified model of YB-1-dependent mechanisms of PARP1 activity regulation. (**a**) Formation of the heteromeric complex of PARP1–YB-1 with damaged DNA. In the ternary complex, YB-1 is a preferable PAR acceptor, but auto-modification of PARP1 occurs too. (**b**) Formation of the complex of YB-1 with PAR covalently attached to PARP1. As soon as the PAR chains on PARP1 reach a certain length, predominant formation of YB-1–PAR rather than YB-1–DNA–PARP1 complexes takes place. (**c**) Upon binding to PAR, YB-1 promotes the formation of shortened PAR during PARP1 auto-PARylation. YB-1 non-covalently binds to PAR chains of auto-PARylated PARP1 and relocates into spatial proximity of the catalytic centre of PARP1. Modified YB-1 molecules dissociate from the complexes, and new unmodified YB-1 molecules bind to the PAR molecule on PARP1.

To sum up the current and previous results, we propose a model of the impact of YB-1 on PARP1 activity (Figure 12).

At a relatively high YB-1/DNA (PARP1) concentration ratio, YB-1 and PARP1 form a ternary complex with DNA, and the efficiency of the complex formation is affected by the type of damaged DNA. Actually, YB-1 contributes to the formation of two types of complexes, one of them is mediated by YB-1 interaction with DNA in the ternary complex with PARP1, and the other by an interaction with PAR during PARP1 activation. Upon binding to DNA, YB-1 forms a heterodimer with PARP1 bound to DNA, and this complex promotes YB-1 trans-PARylation (Figure 12a). If there is an excess of YB-1, then PARylated YB-1 dissociates from this complex and another YB-1 molecule starts a new catalytic cycle of the trans-PARylation reaction in the ternary complex. The subsequent dissociation of PARylated YB-1 increases the turnover of protein PARylation and PAR synthesis. After the binding to PAR during PARP1 automodification, YB-1 by its C-terminal domain is attracted by PAR in the PARP1–DNA complex and inhibits the elongation of the PAR polymer (Figure 12b). This effect is behind the formation of shortened PAR during PARP1 auto-PARylation in the absence of other cation co-factors (Figure 12c). Therefore, YB-1 can block the PAR chain elongation and simultaneously acts as a target of trans-PARylation, suggesting that the binding of YB-1 to PAR occurs in close spatial proximity to the PARP1 catalytic centre. Again, PARylated YB-1 dissociates, increasing the turnover of protein PARylation and PAR synthesis. This model is consistent with the observed extensive trans-PARylation of YB-1 and shortening of the PAR chain during PARP1 auto-PARylation. 

In conclusion, we can hypothesise that the regulation of PARP1 activity by PAR acceptor proteins may change both the extent of PARP1 autoPARylation and the overall protein PARylation level. YB-1, while being PARylated, rapidly dissociates from complexes with PARylated PARP1 (or DNA), and the next YB-1 molecule interacting with PAR (or DNA) initiates the next round of PARylation. The dissociation of PARylated YB-1 from its complexes with activated PARP1 and damaged DNA and subsequent association of the next YB-1 molecule strongly facilitate the reaction turnover. It increases the level of protein PARylation and PAR synthesis. We suppose that this model can be generalized to explain the stimulation of PARP activity by some protein factors cooperating with PARPs [34,35]. 

## Figures and Tables

**Table 1:** Structures and names of the DNA substrates.

Name	Oligodeoxynucleotide Sequences (5′→3′)
ss32	5′-GGCGATTAAGTTGGGAAACGTCAGGGTCTTCC-3′
	
	
ds55	5′-CGGTATCCACCAGGTCTGAGACAACGATGAAGCCCAAGCCAGATGAAATGTAGTC-3′ 3′-GCCATAGGTGGTCCTGACTCTGTTGCTACTTCGGGTTCGGTCTACTTTACATCAG-5′
	
	
mm55	5′-CGGTATCCACCAGGTCTG**A**GACAACGATGAAGCCCAAGCCAGATGAAATGTAGTC-3′ 3′-GCCATAGGTGGTCCTGAC**G**CTGTTGCTACTTCGGGTTCGGTCTACTTTACATCAG-5′
	
	boldfaced nucleotides indicate the position of the A–G mismatch
	
	
Bubble(5)	5′-CGGTATCCACCAGGTC**ACUCT**CAACGATGAAGCCCAAGCCAGATGAAATGTAGTC-3′ 3′-GCCATAGGTGGTCCTG**ACGCT**GTTGCTACTTCGGGTTCGGTCTACTTTACATCAG-5′
	
	boldfaced nucleotides indicate the position of the 5 nt bubble
	
	
Bubble(17)	5′-CGGTATCCAC**GTCCATACUCTGTGTTG**TGA AGCCCAAGCCAGATGAAATGTAGTC-3′ 3′-GCCATAGGTG**GTCCAGACGCTGTTGCT**ACTTCGGGTTCGGTCTACTTTACATCAG-5′
	
	boldfaced nucleotides indicate the position of the 17 nt bubble
	
	
Nick	^ 3′-OH^_\ /_^5′-Phosphate^ 5′-GGCGATAAAGTTGGGAAACGTCAGGGTCTTCC-3′ 3′-CCGCTATTTCAACCCTTTGCAGTCCCAGAAGG-5′
	
Gap (one-nucleotide)	^ 3′-OH^_\ /_^5′-Phosphate^ 5′-GGCGATAAAGTTGGG AACGTCAGGGTCTTCC-3′ 3′-CCGCTATTTCAACCCTTTGCAGTCCCAGAAGG-5′
	
	
3′-Recessed	5′-GGCGATAAAGTTGGG-3′ 3′-CCGCTATTTCAACCCTTTGCAGTCCCAGAAGG-5′
	
	
Dumbbell	T-T-GCTTGAAGGCGCTTCGAAGACGG-T-T | | T-T-CGAACTTCCGCGAAGCTTCTGCC-T-T
	
	
Gap dumbbell (one-nucleotide)	^ 3′-OH^_\ /_^5′-Phosphate^ T-T-GCTTGAAGGCG TTCGAAGACGG-T-T | | T-T-CGAACTTCCGCGAAGCTTCTGCC-T-T
	
	
Nick dumbbell	^ 3′-OH^_\ /_^5′-Phosphate^ T-T-GCTTGAAGGCGCTTCGAAGACGG-T-T | | T-T-CGAACTTCCGCGAAGCTTCTGCC-T-T

**Table 2:** Schematic representation of the 147 bp DNA and mononucleosome substrates used in this study. The sequence of the 23 bp region indicated by the circle is presented. The star denotes the position of the one-nucleotide gap formed after uracil-DNA glycosylase (UDG) and EndoIII treatment.

Name; Sequence in the Circle	Scheme
147 bp DNA 5′-ACCCCAGGGAC**U**TGAAGTAATAA-3′ 3′-TGGGGTCCCTG**A**ACTTCATTATT-5′	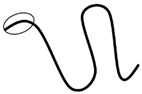
147 bp DNA (gap) ^OH^_\ /_^Phosphate^ 5′-ACCCCAGGGAC TGAAGTAATAA-3′ 3′-TGGGGTCCCTG**A**ACTTCATTATT-5′	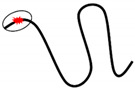
Mononucleosome 5′-ACCCCAGGGAC**U**TGAAGTAATAA-3′ 3′-TGGGGTCCCTG**A**ACTTCATTATT-5′	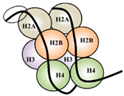
Mononucleosome (gap) ^OH^_\ /_^Phosphate^ 5′-ACCCCAGGGAC TGAAGTAATAA-3′ 3′-TGGGGTCCCTG**A**ACTTCATTATT-5′	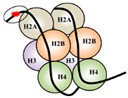

## Supplementary Materials

The following are available online at https://www.mdpi.com/2218-273X/10/9/1325/s1, Figure S1: Mononucleosome reconstitution, Figure S2: Electrophoretic analysis of the pBR plasmid in a 0.75 % agarose gel under non-denaturing conditions with EtBr staining, Figure S3: Analysis of PARP1 activity in the absence or presence of DNA.
