# Supplementary material for: Regulation of Poly(ADP-Ribose) Polymerase 1 Activity by Y-Box-Binding Protein 1
Source: Biomolecules. 2020 Sep 16;10(9):1325. doi: 10.3390/biom10091325 (PMC7565162; doi:10.3390/biom10091325)
Supplement: Supplementary file 1 [file biomolecules-10-01325-s001.pdf]

Supplementary Materials

1. Supplementary Figures

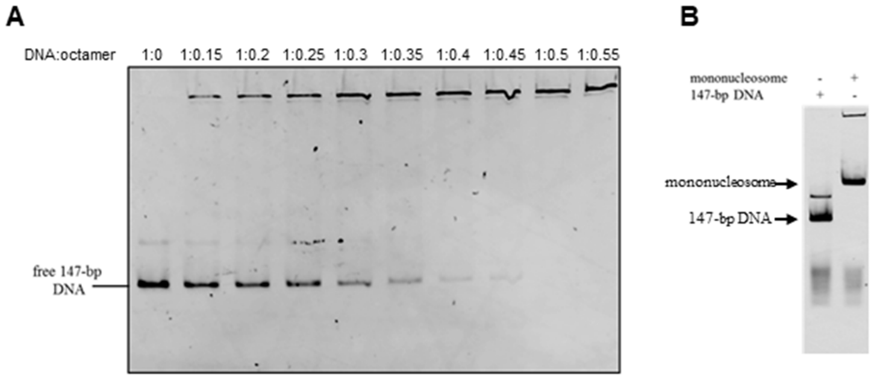

Supplementary Figure S1. Mononucleosome reconstitution. (A) Electrophoretic mobility of 5′-FAM–labelled products after mononucleosome quick-time reconstitution from model FAM-DNA and histone octamers in a 4% polyacrylamide gel under non-denaturing conditions. Samples were supplemented with DNA and octamers at various ratios from 1.00:0.15 to 1.00:0.55 with an increment of 0.05.(B) Electrophoretic mobility of 5′-FAM–labelled products after the process of mononucleosome salt dialysis reconstitution from model FAM-DNA and histone octamers in a 4% polyacrylamide gel under non-denaturing conditions at the 1.00:0.45 ratio of DNA to octamers.

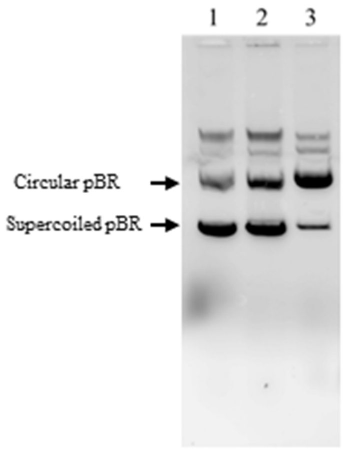

Supplementary Figure S2. Electrophoretic analysis of the pBR plasmid in a 0.75 % agarose gel under non-denaturing conditions with EtBr staining. Lane 1: the intact pBR plasmid, lane 2: the pBR plasmid after sodium citrate treatment, lane 3: the pBR plasmid after sodium citrate and APE1 treatment.

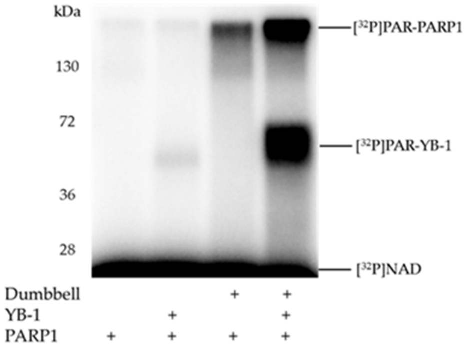

Supplementary Figure S3. Analysis of PARP1 activity in the absence or presence of DNA. Protein PARylation in the absence or presence of dumbbell DNA and [^{32}P]NAD according to SDS-PAGE and phosphor imaging. The reaction mixtures contained 100 nM PARP1, 1600 nM YB-1 (where indicated) 4 μM NAD+ and [^{32}P]NAD (0.4 μCi) and 100 nM DNA substrate (where indicated). The mixtures were incubated at 37°C for 15 min, the reactions were stopped by adding SDS sample buffer.
